# Supplementary material for: The vestibular calyceal junction is dismantled following subchronic streptomycin in rats and sensory epithelium stress in humans
Source: Arch Toxicol. 2023 May 17;97(7):1943–61. doi: 10.1007/s00204-023-03518-z (PMC10256663; doi:10.1007/s00204-023-03518-z)
Supplement: Supplementary file 3 — Supplementary file3 (PPTX 21709 KB) [file 204_2023_3518_MOESM3_ESM.pptx]

## Slide 1
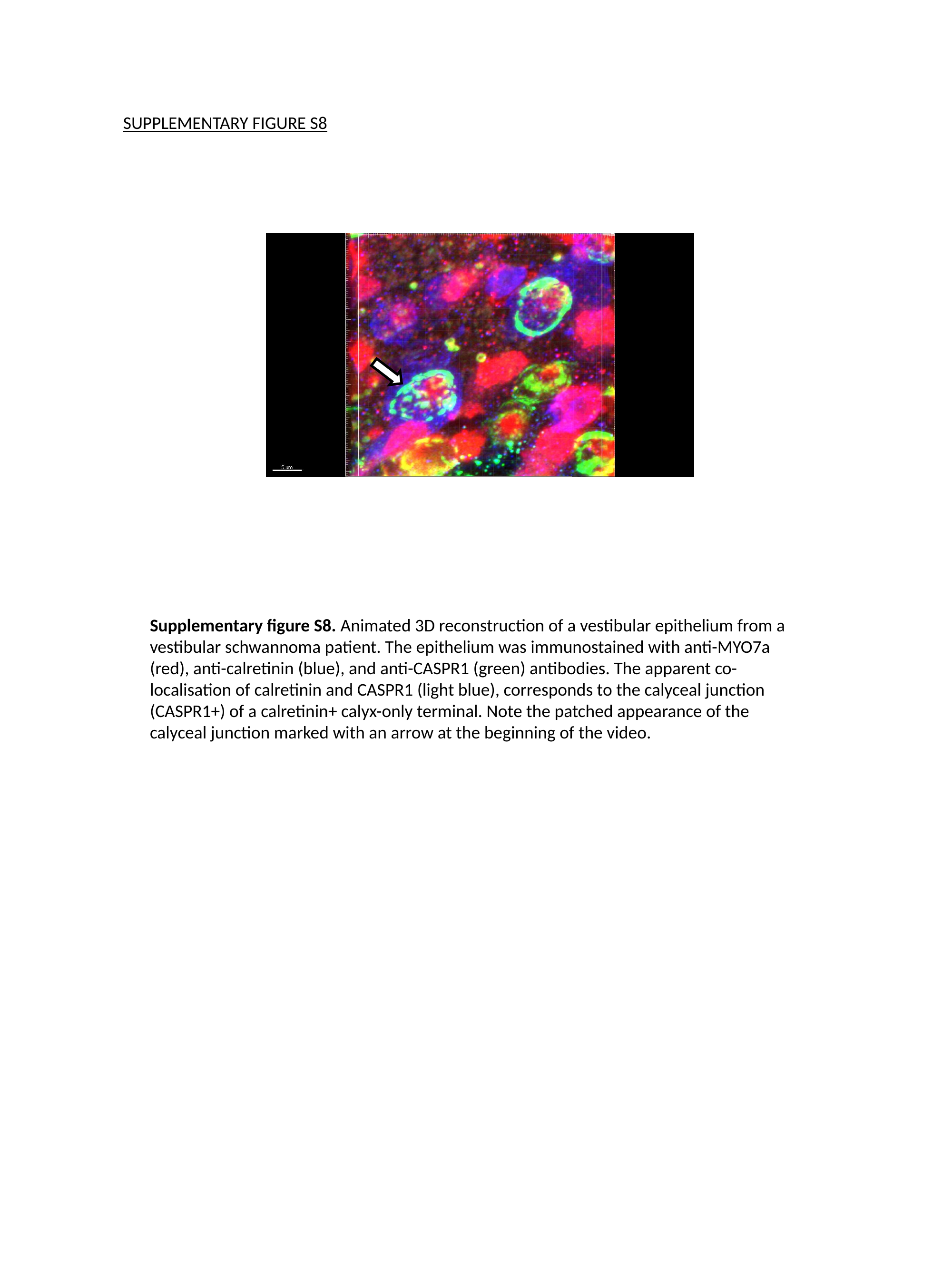

SUPPLEMENTARY FIGURE S8
Supplementary figure S8. Animated 3D reconstruction of a vestibular epithelium from a vestibular schwannoma patient. The epithelium was immunostained with anti-MYO7a (red), anti-calretinin (blue), and anti-CASPR1 (green) antibodies. The apparent co-localisation of calretinin and CASPR1 (light blue), corresponds to the calyceal junction (CASPR1+) of a calretinin+ calyx-only terminal. Note the patched appearance of the calyceal junction marked with an arrow at the beginning of the video.
